# Drug capture materials based on genomic DNA-functionalized magnetic nanoparticles

**DOI:** 10.1038/s41467-018-05305-2

**Published:** 2018-07-20

**Authors:** Carl M. Blumenfeld, Michael D. Schulz, Mariam S. Aboian, Mark W. Wilson, Terilynn Moore, Steven W. Hetts, Robert H. Grubbs

**Affiliations:** 1 Arnold and Mabel Beckman Laboratories for Chemical Synthesis, Division of Chemistry and Chemical Engineering, California Institute of Technology, Pasadena, CA 91125 USA; 2 Interventional Radiology Research Laboratory, Department of Radiology and Biomedical Imaging, University of California-San Francisco, San Francisco, CA 94143 USA

## Abstract

Chemotherapy agents are notorious for producing severe side-effects. One approach to mitigating this off-target damage is to deliver the chemotherapy directly to a tumor via transarterial infusion, or similar procedures, and then sequestering any chemotherapeutic in the veins draining the target organ before it enters the systemic circulation. Materials capable of such drug capture are yet to be fully realized. Here, we report the covalent attachment of genomic DNA to iron-oxide nanoparticles. With these magnetic materials, we captured three common chemotherapy agents—doxorubicin, cisplatin, and epirubicin—from biological solutions. We achieved 98% capture of doxorubicin from human serum in 10 min. We further demonstrate that DNA-coated particles can rescue cultured cardiac myoblasts from lethal levels of doxorubicin. Finally, the in vivo efficacy of these materials was demonstrated in a porcine model. The efficacy of these materials demonstrates the viability of genomic DNA-coated materials as substrates for drug capture applications.

## Abstract (web summary)

Chemotherapy agents are prone to producing severe side-effects, and their sequestration prior to their entering of the circulatory system is thus highly desirable. Here, the authors functionalize iron oxide nanoparticles with genomic DNA and achieve sequestration of doxorubicin, cisplatin, and epirubicin from biological solutions.

## Introduction

The systemic toxicity of chemotherapy is a widely recognized problem in oncology. Off-target damage often persists indefinitely, adversely affects patient survival, and restricts dose and treatment options^1,2^. Direct administration of chemotherapy agents to the tumor via transarterial chemoembolization (TACE), or similar procedures, followed by sequestration of any chemotherapeutic that enters systemic circulation would mitigate this damage if materials capable of such drug capture were fully realized^3,4^.

Hepatocellular carcinoma (HCC) is the third leading cause of cancer-related deaths worldwide^5^. Liver transplantation is the most definitive approach for treatment; however, less than 30% of HCC patients are eligible^6^. Direct delivery of drug to a tumor via intraarterial chemotherapy (IAC) and its variant, TACE, is often used as a bridge to transplantation, shrinking HCC or at least controlling its growth through recurrent treatments until curative transplant is possible. In cases where surgery is untenable, chemotherapy is often the only recourse. Targeted therapy, however, does not completely eliminate side-effects.

**Fig. 1:**
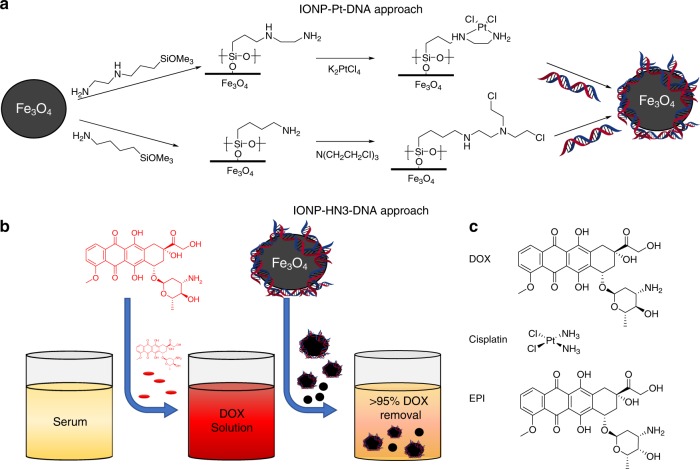
Experimental approach. **a** Two synthetic approaches for covalently attaching genomic DNA onto iron-oxide nanoparticle (*IONP*) surfaces. **b** Drug capture concept. **c** Three common chemotherapy agents used in this study

Three of the most common drugs used to treat HCC are doxorubicin (DOX), epirubicin (EPI), and cisplatin (Fig. 1c), all of which act on DNA^7^. DOX and EPI function by intercalating between DNA base pairs, while cisplatin is a DNA crosslinker that functions by binding to guanine^8,9^. A major problem for these anticancer compounds is toxicity in non-targeted tissues. DOX and EPI toxicity can result in cardiomyopathy and congestive heart failure^8–10^. Similarly, cisplatin elicits side-effects including extensive nephrotoxicity and neurotoxicity^11,12^. To reduce the likelihood of cardiac toxicity, cumulative dosage of DOX is generally limited by clinicians to 400–450 mg/m^2^, though lower cumulative dosages (300 mg/m^2^) are known to increase the risk of congestive heart failure^13,14^. Still, a single standard dose of DOX (50–75 mg) can result in severe side-effects, yet higher dosages of DOX are known to be associated with greater tumor suppression. Consequently, a balance must be struck in order to maximize drug dose, leading to better tumor suppression, while simultaneously avoiding catastrophic off-target toxicity. Although limiting a patient’s lifetime cumulative dose is the most effective way to avoid cardiotoxicity, this approach necessarily limits anti-cancer efficacy^15^.

The unwanted systemic toxicity of chemotherapy agents has inspired a number of more targeted approaches. One such approach is TACE, during which liver blood flow is occluded in conjunction with administration of high dose chemotherapy directly to the tumor^3,16^. Both during TACE and after liver blood flow is restored, however, up to 50% of residual chemotherapeutics enter systemic circulation and cause off-target toxicity^17^.

Efforts have been made toward reducing non-targeted toxicity during TACE. In 2014 Patel and coworkers proposed chemotherapy filtration devices (“ChemoFilters”) that employed sulfonated ion-exchange resins with affinity for DOX. Such a device could be deployed via catheter in the hepatic vein, “downstream” from the site of chemotherapy administration, where it can intercept any residual chemotherapy agents before they reach the heart and enter systemic circulation. They demonstrated a 52% reduction in DOX concentration from porcine serum over 10 min, and showed that such a device could be successfully deployed during a simulated TACE procedure^3^. In 2016, the ChemoFilter approach inspired the development of more elaborate block copolymer membranes for DOX capture, which achieved up to 90% removal of DOX in 31 min from phosphate buffered saline (PBS)^4^.

A ChemoFilter device is intended to be placed downstream of blood outflow from the tumor that is being treated with intra-arterial chemotherapeutics. In the case of HCC, which is located in the liver, the ChemoFilter would be placed into the suprahepatic inferior vena cava (IVC) immediately prior to administration of IAC into the hepatic artery and then removed shortly after drug capture has been achieved^3,18^. Here we report the development of a drug-capture material based on genomic DNA and iron oxide particles. In vitro studies confirm that these materials can rescue cells from the toxic effects of DOX. A ChemoFilter device is constructed from this material that rapidly removes chemotherapy agents from relevant biological solutions, including human serum and porcine blood, and in vivo studies confirm that DOX can be removed from the bloodstream by an intraarterial device constructed from these iron oxide/genomic DNA materials.

## Results and discussion

### Material design and synthesis

Inspired by the ChemoFilter concept, we designed and synthesized DNA-functionalized materials based on magnetite (Fe_3_O_4_) nanoparticles, capable of rapidly capturing chemotherapy agents. Central to our approach is the direct covalent attachment of genomic DNA. Functionalizing surfaces with DNA has historically involved tagging either the backbone or bases of synthetic DNA with an appropriate moiety, or attaching the DNA via a reactive end-group. These approaches are highly useful and enable complete control of the DNA sequence used resulting in the development of numerous interesting materials^19–26^; however, they are limited by the relatively high cost of synthetic DNA. The synthesis of large amounts of such materials would be prohibitively expensive for most applications.

Functionalization with genomic DNA is an alternative approach that may be appropriate for certain applications; however, this approach is relatively unexplored. Pierre and coworkers recently synthesized magnetic nanoparticles with surface-bound intercalating groups, and showed that such materials can bind to genomic DNA^27^. To our knowledge, however, no one has reported the covalent attachment of genomic DNA to a surface. Here, we report two methods of attaching genomic DNA to nanoparticles, both on multi-gram scale (Fig. 1a). We show that the resulting materials are capable of removing DNA-targeting chemotherapy agents from solution both rapidly and in the presence of potential biological intereferents (e.g., serum proteins and other blood components).

DNA-alkylating agents are a common motif in chemotherapy. By forming covalent crosslinks between DNA strands, these drugs prevent the DNA from being accurately duplicated, ultimately leading to apoptosis. To attach genomic DNA to magnetic nanoparticles, we used an approach analogous to DNA-alkylating/crosslinking drugs (Fig. 1a). The first approach was inspired by cisplatin. To synthesize IONP-Pt-DNA samples, the hydroxylated surface of Fe_3_O_4_ was silylated with N-(2-aminoethyl)-3-aminopropyltrimethoxysilane exposing a chelating diamine functionality. This sample was treated with an excess of potassium tetrachloroplatinate to create an analog of cisplatin by which DNA could be anchored to the surface. Cisplatin’s cytotoxicity is thought to stem from its coordination with nucleophilic N7-sites of purine bases, resulting in crosslinks^28^. We hoped to accomplish DNA crosslinking to the surface through this mechanism. The sample was then exposed to DNA to produce IONP-Pt-DNA.

The second approach was modeled on nitrogen mustard chemotherapy agents. IONP-HN3-DNA samples were prepared first by functionalizing Fe_3_O_4_ with 4-aminobutyltriethoxysilane to install free amines on the surface. This particle was then treated with excess tris(2-chloroethyl)amine hydrochloride (HN3·HCl) to create a scaffold for DNA functionalization. HN3·HCl, the hydrochloride salt of the nitrogen mustard HN3, undergoes aziridinium formation when deprotonated, and is attacked readily by the nucleophilic moieties of DNA^29^. The functionalized particle was exposed to DNA resulting in IONP-HN3-DNA. Both materials were characterized by scanning electron microscopy, electron dispersive scattering (EDS), elemental analysis, and infrared spectroscopy (see Supplementary Figs. 2& 3 and 11–24, and Supplementary Table 1). Microscopy images of the particles in solution revealed significant aggregation resulting in an average particle diameter of 4.2 μm with several larger (>10 μm) aggregates. Elemental analysis indicated that these aggregates were 18% DNA by mass in the case of IONP-HN3-DNA and 14.7% DNA by mass in the case of IONP-Pt-DNA.

### In vitro testing in simple solutions, serum, and blood

**Fig. 2:**
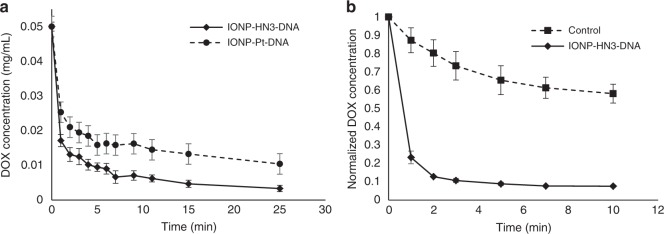
DOX capture in human serum and porcine blood. **a** Decrease in DOX concentration in human serum, determined by fluorescence, as a result of DOX capture by IONP-HN3-DNA and IONP-Pt-DNA; 100 ± 5 mg particle in 20 mL (0.05 mg/mL), 1 mg total DOX, 37 °C; error bars = 1 standard deviation (*n* = 3). **b** Decrease in DOX plasma concentration as a result of DOX capture by IONP-HN3-DNA from porcine whole blood; 100 ± 5 mg IONP-HN3-DNA in 20 mL (0.05 mg/mL), 1 mg total DOX, 37 °C; error bars = 1 standard deviation (*n* = 3)

In order to evaluate the efficacy of our materials at scavenging chemotherapy agents from solution we studied DOX-binding in PBS and human serum at 37 °C to approximate the biological environment in which these materials would have to operate (Fig. 1b). We found that IONP-HN3-DNA was able to capture 93% of DOX, on average, from a 0.05 mg/mL solution of human serum in 25 min, while IONP-Pt-DNA averaged 79% (Fig. 2a). In both cases, the kinetics were extremely rapid, with about 50% of DOX capture occurring within 1  min in the case of IONP-Pt-DNA and over 65% DOX capture occurring within 1 min for IONP-HN3-DNA. Based on these results, we carried out all further tests with IONP-HN3-DNA.

Interestingly, both materials were highly effective, despite the known binding of DOX with serum albumin. It is known that DNA intercalation is the kinetically more favorable process^30,31^, and we believe that this kinetic advantage enabled our material to capture DOX from serum solution, despite the thermodynamics being in favor of serum binding overall. We posit that over longer timescales, serum binding would be the dominant process; however, since TACE is a relatively short procedure (<1 h), we believe that kinetic factors will dominate in the performance of any material or device.

Drug capture was also evaluated in porcine whole blood, by measuring DOX plasma concentration over time. We observed some DOX removal due to binding to the non-plasma blood components, which we cannot deconvolute from capture by our materials. Nevertheless, there is rapid reduction of DOX concentration in the blood plasma within 1 min after exposure to our material, reaching a 92% reduction in DOX plasma concentration over 10 min, in stark contrast to the control experiment (Fig. 2b). This experiment conclusively demonstrates that our materials are capable of capturing DOX from whole blood.

**Fig. 3:**
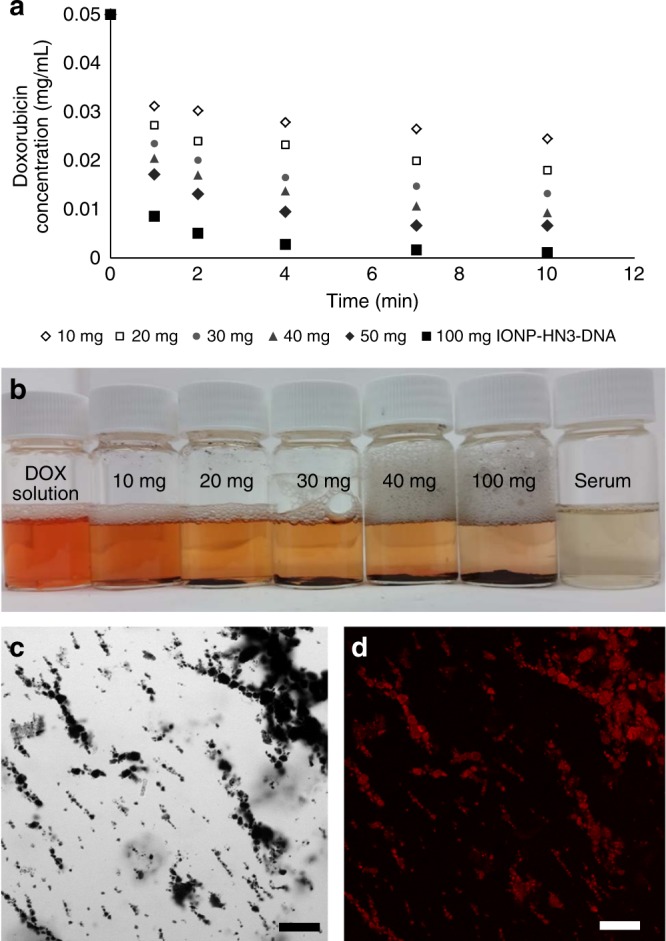
DOX capture with IONP-HN3-DNA particles. **a** DOX capture as a function of the amount of IONP-HN3-DNA from a DOX serum solution (0.5 mg total DOX, 0.05 mg/mL); average of three experiments, data set with error bars in Supplementary Fig. 6. **b** Resulting serum solutions from experiments summarized in **a**. **c** Brightfield image of IONP-HN3-DNA aggregates bound to DOX; scale bar = 50 μm. **d** Fluorescence from DOX bound to IONP-HN3-DNA; scale bar = 50 μm

To better understand the DOX-capture capacity of IONP-HN3-DNA, we performed a series of experiments in which nanoparticle loading was systematically varied (Fig. 3a, b, further data in the Supplementary Information). These experiments revealed a roughly linear trend in DOX-capture as a function of the amount of particle added, up to a plateau around 100 mg material added per mg DOX, resulting in ~90% DOX capture in 10 min. Further DOX capture appears less favorable after this point. We believe this plateau is the result of competition with serum binding, which makes that portion of DOX unavailable for capture by our particles, as well as the typical kinetic effects of diminishing concentration. The absorption of DOX onto the particles was further verified by performing confocal fluorescence microscopy (Fig. 3c and d). This technique allowed visualization of the fluorescence of DOX bound to the surface of the particles.

Our approach is general for all DNA-targeting chemotherapy agents. To demonstrate this fact, we performed further experiments on two additional common DNA-targeting chemotherapeutics, cisplatin and EPI. We performed an initial cisplatin-binding experiment in PBS solution with IONP-HN3-DNA and monitored the decrease of cisplatin concentration by inductively coupled plasma-mass spectrometry (ICP-MS). Approximately 20% of the cisplatin was captured from solution over 30 min, with little improvement over longer time periods (see Supplementary Fig. 4). We confirmed the presence of captured cisplatin on the surface of the particles by x-ray photoelectron spectroscopy. We believe these relatively low levels of drug capture are due to cisplatin not being in the aquo state, which happens intracellularly and is necessary in order to bind to DNA. Because the particles were not highly effective for cisplatin capture in PBS, we did not perform further experiments in more complex media such as serum or blood.

Along with DOX and cisplatin, EPI is among the most commonly used chemotherapeutic agents for treating HCC. We evaluated the efficacy of our materials for capturing EPI using a set of experiments analogous to those we used with DOX (see Supplementary Fig. 5). Our particles were highly effective at sequestering EPI from serum, with 68% captured after 25 min. The sequestered amount would lead to a reduction in unwanted side-effects if achieved in vivo.

### In vitro evaluation of biological efficacy

**Fig. 4:**
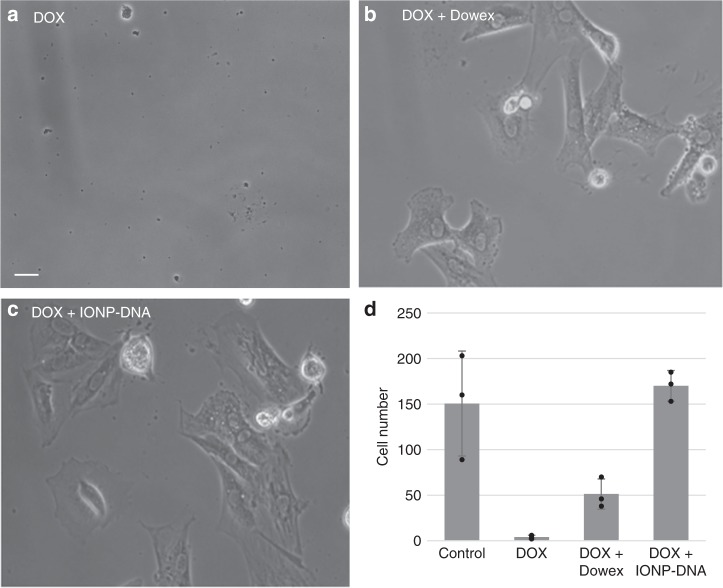
IONP-HN3-DNA rescue cultured cells from DOX toxicity (scale bar = 5 μm). **a** Rat heart myoblasts H9C2 cultured with DOX (0.05 mg/mL)—myoblasts are killed by adding DOX to culture (DOX, **d**). **b** Myoblasts treated with DOX (0.05 mg/mL) and 0.19 g Dowex ion-exchange resin beads—myoblasts are partially rescued ((DOX + Dowex, **d**). **c** Myoblasts treated with DOX (0.05 mg/mL) and 0.19 g IONP-HN3-DNA—myoblasts are completely rescued (DOX + IONP-DNA, **d**). **d** Cell counts for each experimental condition; error bars = 1 standard deviation (*n* = 3)

The ability of IONP-HN3-DNA to detoxify DOX was tested in vitro in an H9C2 rat heart myoblast cell culture assay (Fig. 4). These experiments demonstrated that DNA-coated particles could rescue cultured cardiac myoblasts from lethal levels of DOX more effectively than the ion exchange resin Dowex, which itself had been previously shown to reduce levels of DOX in vivo^3^.

### In vivo evaluation of drug capture

**Fig. 5:**
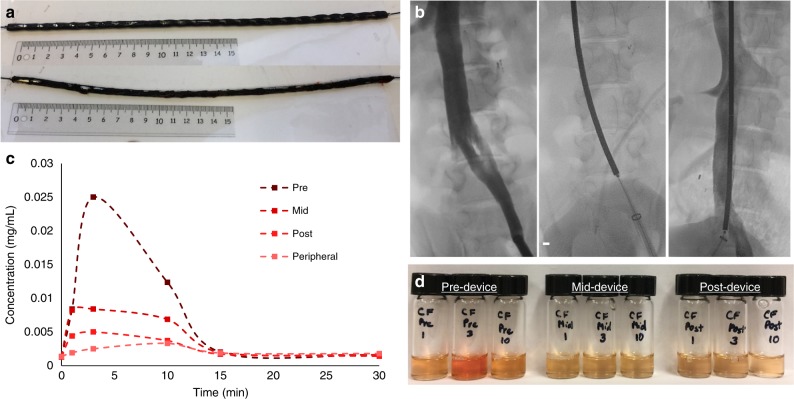
In vivo results. **a** Device containing 25 magnets (1 cm × 0.5 cm) with IONP-HN3-DNA coating (above), and the same device after the in vivo experiment (below), demonstrating minimal loss of particles after removal of the device. **b** Fluoroscopy images during in vivo porcine experiment demonstrating the inferior vena cava with opacified right renal veins (scale bar = 5 mm). The device was placed within the inferior vena cava. The sampling catheters were placed immediately proximal to the device, prior to the renal vein, and distal to the device. **c** DOX concentration measurements from pre-device, mid-device, post-device, and peripheral locations. **d** Plasma solutions from the experiment described in **c**: Left 3, pre-filter samples taken at 1, 3, and 10 min; Center 3, mid-filter samples taken at 1, 3, and 10 min; Right 3, post-filter samples taken at 1, 3, and 10 min

A device (Fig. 5a) consisting of IONP-HN3-DNA magnetically adhered to the surface of cylindrical rare-earth magnets strung along a PTFE-coated nitinol wire was evaluated using a closed loop flow model^3^ (see Supplementary Fig. 25) and subsequently tested in vivo using a porcine model. The device was inserted into the IVC and DOX was injected over 10 min at a rate of 2.5 mL/min into the left common iliac vein proximal to the device (Fig. 5b). As the drug flowed through the IVC, it made contact with the IONP-HN3-DNA adherent to the surface of the device and was captured. Blood aliquots were taken proximal to (upstream), adjacent to the midpoint of, and distal to (downstream from) the device using separate catheters. Peak DOX concentration was observed at 3 min, since the blood at the injection site had recirculated and live injection was still underway. At 3 min (peak concentration), a 60% reduction in serum DOX concentration was observed half-way across the device, while a total reduction of 82% was observed at the end of the device (Fig. 5c, d).

In conclusion, we have demonstrated two viable synthetic pathways to genomic DNA-functionalized magnetic particles, both on multi-gram scale. Moreover, these methodologies for DNA surface functionalization are not limited to magnetic metal oxides, but may also be exploited for other substrates. The synthesized materials captured three commonly used chemotherapy agents from relevant biological solutions (PBS, human serum, or porcine whole blood), at therapeutically relevant concentrations and timescales. A proof of concept device was developed, which demonstrated efficient capture of DOX in vivo. Similar devices could be readily developed that would potentially reduce the off-target toxicity and damaging side-effects associated with the use of DOX, cisplatin, and EPI during TACE or similar procedures. Ultimately, we believe our approach is general for all DNA-targeting chemotherapy drugs, and while further development is needed, we hope that this work will provide a foundation for future work on DNA-based materials and drug capture approaches both for oncologic and non-oncologic applications.

## Methods

### Instrumentation

Fluorescence measurements were made using a 96-well plate on a Molecular Devices FlexStation 3 Multi-mode microplate reader. Scanning electron micrographs (SEM), as well as EDS measurements were made on a Zeiss 1550VP field emission SEM equipped with an Oxford EDS module. ICP-MS was carried out on an HP 4500 ICP-MS equipped with a Cetac ASX-500 autosampler. Infrared measurements were made on a Nicolet iS50 Fourier transform infrared spectrometer equipped with a DuraScope ATR unit. C, H, N analyses were carried out using a PerkinElmer 2400 Series II CHN Elemental Analyzer. Fluorescence microscopy was performed on an inverted laser scanning confocal Zeiss LSM 710 microscope equipped with an argon laser and photomultiplier tube detector, and particle size was determined by image analysis of at least 100 particles measured on their widest dimension.

### General procedures

Unless otherwise stated reactions were carried out on the bench. Fe_3_O_4_ (40 nm APS, 99%) was purchased from Nanostructured & Amorphous Materials, Inc. Silane reagents were purchased from Gelest, Inc. Genomic DNA (isolated from Herring sperm), human serum (OptiClear), H9C2 rat heart myoblasts, and cisplatin were purchased from Sigma Aldrich. DOX was purchased from LC Labs and EPI was purchased from Biotang Inc. Potassium tetrachloroplatinate was purchased from Pressure Chemicals. All reagents not otherwise mentioned were purchased from Sigma Aldrich, and were used without further purification.

### Device construction

Twenty-five cylindrical rare-earth magnets (N52 grade, 5 mm OD × 1 mm ID × 5 mm L, magnetized through the diameter) were strung along the length of a PTFE-coated nitinol wire (Terumo Glidewire). IONP-HN3-DNA (1.0 g) was suspended in water and subsequently magnetically adhered to the surface of this device.

### Flow model experiments

A closed-circuit flow model was used to measure DOX clearance in a setting that simulates suprahepatic IVC conditions^3,32^. In this model, the porcine blood is circulated through the polyvinyl chloride tubing at a rate of ∼150 mL/min. The tubing size matches the average human hepatic vein measuring 1.2 cm as described previously^18^. Testing was performed with 200 mL porcine blood and samples were obtained from the tubing downstream from the device.

### In vitro experiments

H9C2 rat heart myoblasts (procured from Sigma Aldrich) were cultured in well plates (four replicates per condition) for 48 h after which cells were imaged with a light microscope and counted. In the control experiment, cells were cultured in RPMI medium with 10% fetal bovine serum. In the DOX experiment, cells were cultured with 0.05 mg/mL DOX added to the medium. In the cell rescue experiments, 0.19 g of either Dowex ion-exchange resin or IONP-HN3-DNA was added to the culture media prior to introduction of DOX (final DOX concentration: 0.05 mg/mL).

### In vivo porcine experiments

In vivo device testing was performed in farm swine (*n* = 1, 45–50 kg), which was under humane care. Experimentation was under compliance with UCSF IACUC protocols. The animal was monitored with blood pressure, pulse oximetry, heart rate, and electrocardiogram while under general anesthesia with isoflurane. Using fluoroscopic guidance, an 18Fr sheath was placed into the left external iliac vein for introduction of the device. A pre-device sampling catheter was introduced through the right external iliac vein with the tip terminating in the left common iliac vein near the bifurcation. An additional catheter was introduced via the right internal jugular vein with the tip distal to the device in the IVC (post-device). The mid-device catheter and peripheral catheters were introduced through the left internal jugular vein. Prior to the start of the experiments, patency of the venous system was demonstrated using contrast injection (Omnipaque). DOX was injected over 10 min at a rate of 2.5 mL/min into the left common iliac vein proximal to the magnetic device. The pre-device DOX concentration was measured by sampling with a 5 Fr catheter downstream of the DOX infusion. Blood aliquots were taken proximal to, adjacent to the midpoint of, and distal to the device using separate catheters. To clear the sampling catheters, 2 mL of blood was drawn immediately prior to taking the aliquot (3 mL). The blood samples were placed on ice until they were centrifuged to isolate the plasma fraction for analysis. A control experiment was also performed using the same procedures but with no device inserted.

### Particle synthesis

IONP-Pt: 3.31 g Fe_3_O_4_ was dried in vacuo at 120 °C. Upon cooling, the sealed material was introduced into an inert atmosphere nitrogen glovebox. To the Fe_3_O_4_ was added 23 mL anhydrous toluene along with 4 mL N-(2-aminoethyl)-3-aminopropyltrimethoxysilane. The reaction was mechanically stirred on the bench at 110 °C for 2 h and subsequently dried in vacuo at 110 °C for 20 h. The reaction mixture along with 1.0 g K_2_PtCl_4_, was stirred at 70 °C for 21 min and then washed three times with water. Following this, the mixture was diluted to a total volume of 450 mL with 18.2 MΩ water was treated with 1.3 g KCl and an additional 10 mL water.

IONP-Pt-DNA: IONP-Pt materials along with 5.1 g deoxyribonucleic acid from herring sperm were mechanically stirred in 450 mL 18.2 MΩ water at 37 °C for 20 h. To ensure covalent attachment as opposed to being physically adsorbed, the particles were isolated from the reaction mixture, washed three times under vigorous mechanical stirring with 18.2 MΩ water (400 mL) in order to remove unbound DNA, frozen, and lyophilized to afford 3.08 g IONP-Pt-DNA (79% yield based on elemental analysis of DNA content).

IONP-NH2: 4.2 g Of Fe_3_O_4_ was dried in vacuo at 120 °C. The Fe_3_O_4_ was allowed to cool to room temperature under vacuum. To the Fe_3_O_4_ was added to 25 mL toluene (freshly dried over magnesium sulfate) and 3.2 mL 4-aminobutyltriethoxysilane. The reaction was sealed and stirred mechanically for 2 h at 120 °C. The reaction was removed from heat and the particles were isolated from the toluene solution. The reaction mixture was washed once with toluene and subsequently dried in vacuo at 120 °C for 1 h and 45 min. 4.02 g of IONP-HN3 was isolated.

IONP-HN3-DNA: 3.4750 g IONP-HN3 was added to a vial along with 1.02 g HN3·HCl and dimethylformamide (30 mL). The reaction was stirred mechanically for 1 h at room temperature at which point, the particles were isolated from the dimethylformamide. The particles were then washed three times with dimethylformamide. The isolated particle as well as 3.35 g deoxyribonucleic acid from herring sperm were transferred into a flask along with 400 mL 18.2 MΩ water. The reaction was mechanically stirred at 38 °C for 17 h and 45 min. To ensure covalent attachment and to remove any unbound DNA, the particles were then washed thoroughly under vigorous mechanical stirring three times with 18.2 MΩ water (400 mL) and magnetic separation. The particles were then frozen in liquid nitrogen and lyophilized to afford 3.79 g of IONP-HN3-DNA (89% yield as calculated above).

### Representative binding studies

DOX: To a scintillation vial was added 19 mL human serum. Drug was injected at a concentration of 1 mg/mL from a concentrated stock, to bring the total concentration to ∼0.05 mg/mL. An initial time point is taken before drug capture. DNA particles (100 ± 5 mg) were added to the serum mixture, which is constantly, mechanically stirred. 20 s before a time point is taken, a strong, rare earth, magnet is used to isolate the particles at which point a 100 μL aliquot is taken and placed in a 96-well microplate. The solutions are then measured by way of fluorescence on a microplate reader.

Cisplatin: Phosphate-buffered saline solution (19 mL) was added to a scintillation vial. Cisplatin solution (1 mL, 1 mg/mL solution) was then injected, followed by 117 ± 5 mg of IONP-HN3-DNA, and the mixture was mechanically stirred over the course of an hour. At predetermined time points the magnetic materials were temporarily isolated using an external magnet so that 100 μL aliquots could be taken, which were diluted 200× in 2% nitric acid solution and subsequently analyzed by ICP-MS to determine the concentration of platinum remaining in solution.

EPI: Human serum (19 mL) was added to a scintillation vial. EPI solution in water (1 mL, 1 mg/mL solution) was then added. The particles (100 ± 5 mg IONP-HN3-DNA) were then added and the solution was mechanically stirred over the course of 25 min. At predetermined time points, the magnetic materials were temporarily isolated using an external magnet and 100 μL aliquots were taken, which were subsequently diluted 100× in water and analyzed by fluorescence on a microplate reader in order to characterize the amount of EPI remaining in solution.

### Data availability

All data supporting the findings of this study are available within the article and its Supplementary Information. All other data are available from the corresponding author upon reasonable request.

## Electronic supplementary material

Supplementary Information
