# Supplementary material for: Drug capture materials based on genomic DNA-functionalized magnetic nanoparticles
Source: Nat Commun. 2018 Jul 20;9:2870. doi: 10.1038/s41467-018-05305-2 (PMC6054622; doi:10.1038/s41467-018-05305-2)
Supplement: Supplementary file 1 — Supplementary Information [file 41467_2018_5305_MOESM1_ESM.pdf]

Supplementary Information

Drug capture materials based on genomic DNA-functionalized magnetic nanoparticles

Blumenfeld et al.

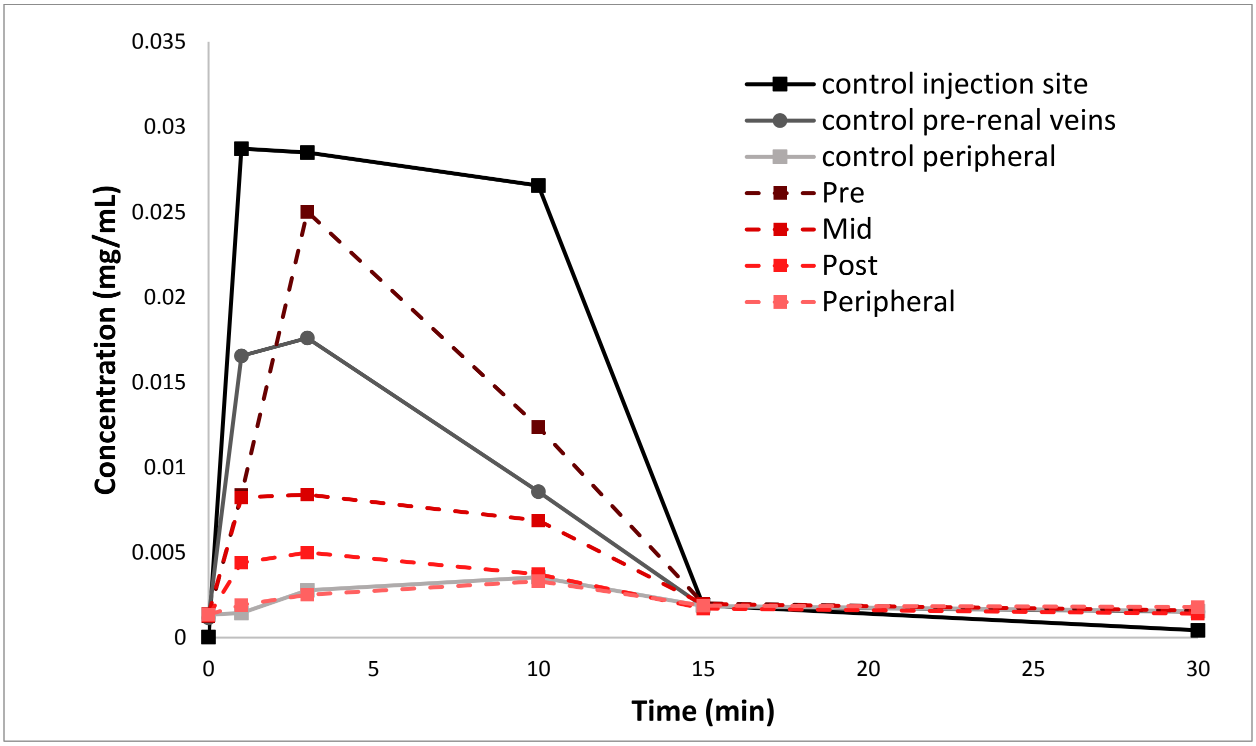

Supplementary Figure 1. ***In-vivo*** **capture data for both control (no device) and IONP-HN3-DNA coated device.**

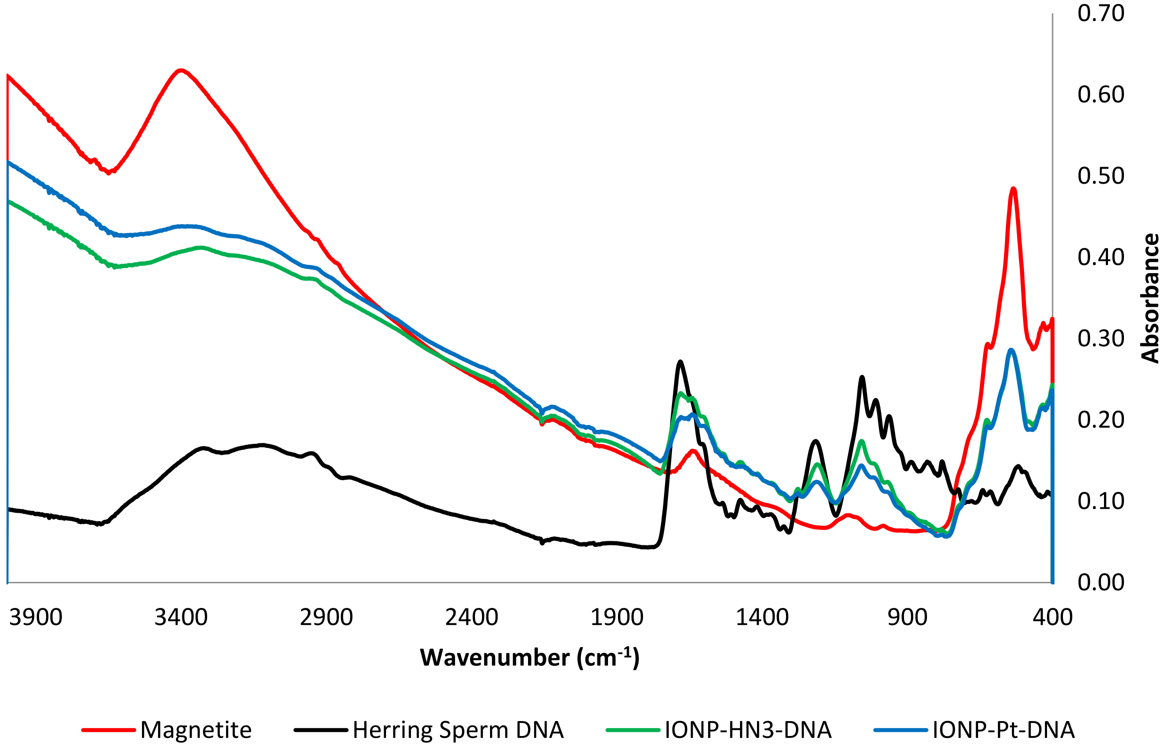

Supplementary Figure 2. Full ATR-IR spectrum of magnetite, genomic DNA, and DNA-functionalized particles (IONP-HN3-DNA and IONP-Pt-DNA).

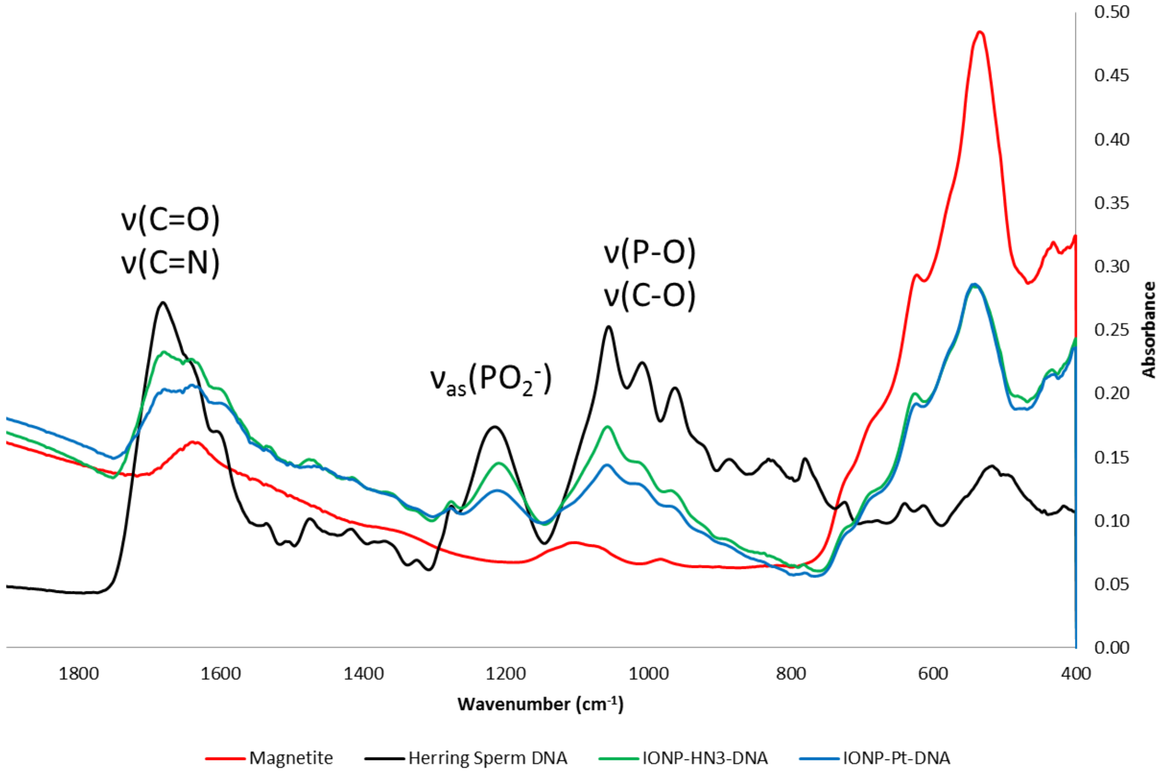

Supplementary Figure 3. ATR-IR spectrum (1800-400 cm^{-1}) of magnetite, genomic DNA, and DNA-functionalized particles [IONP-HN3-DNA and IONP-Pt-DNA].[1]

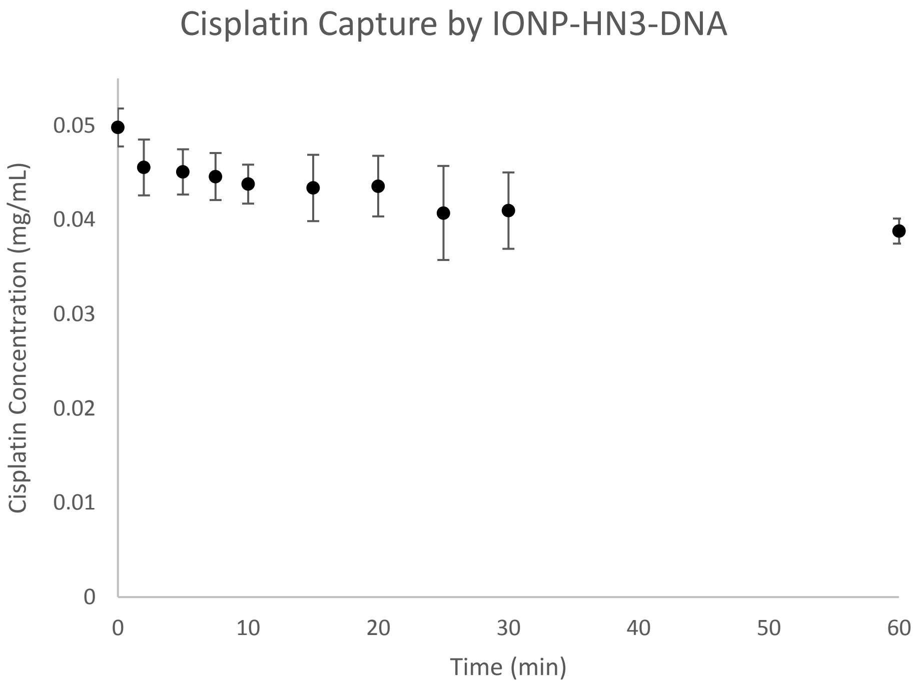

Supplementary Figure 4. Reduction of cisplatin concentration over time due to capture by IONP-HN3-DNA particles, as characterized by ICP-MS. Cisplatin solution (20 mL, 0.05 mg/mL) with 100 mg IONP-HN3-DNA. Average of three runs (error bars = 1 standard deviation).

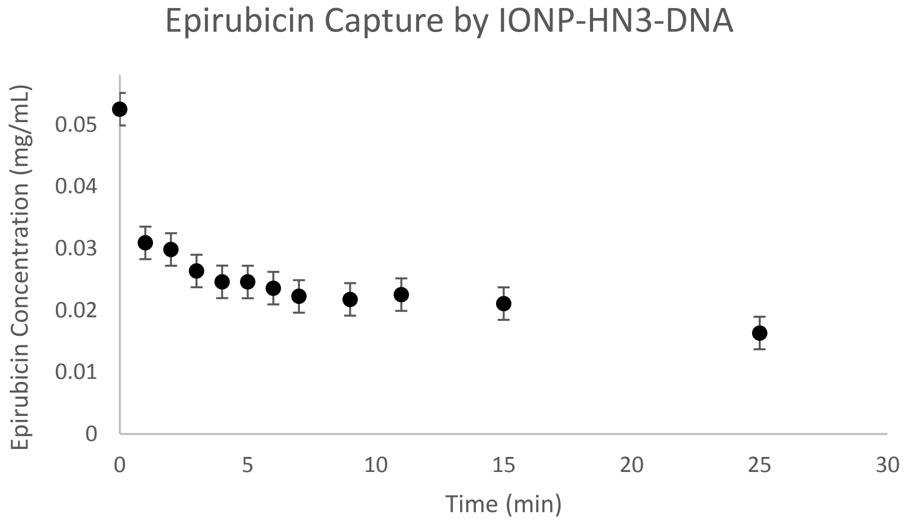

Supplementary Figure 5. Reduction of EPI concentration over time due to capture by IONP-HN3-DNA as characterized by fluorescence measurements. EPI solution (20 mL, 0.05 mg/mL) with 100 mg IONP-HN3-DNA. Average of three runs (error bars = 1 standard deviation).

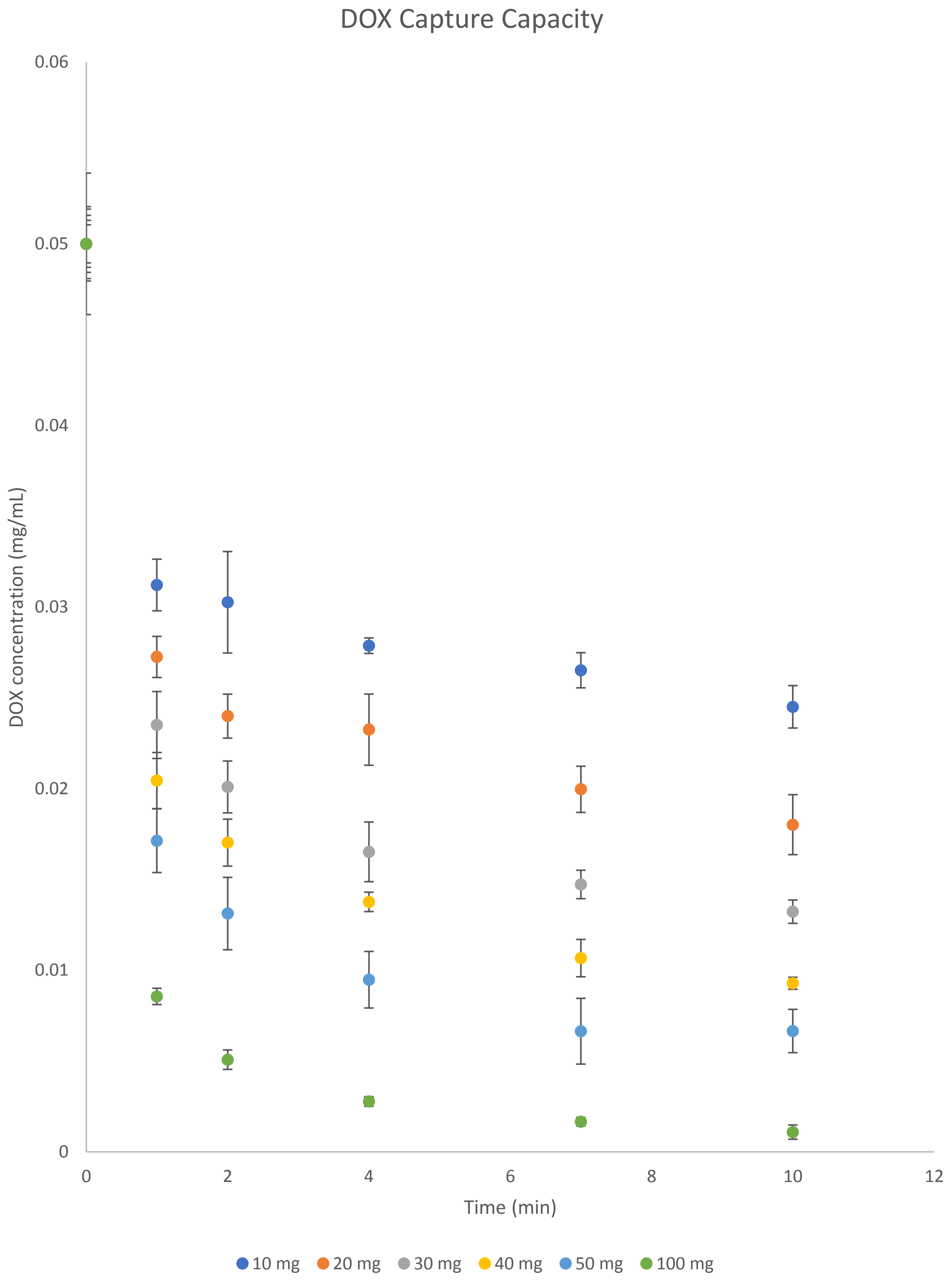

Supplementary Figure 6. DOX capture as a function of particle loading. Average of three runs (error bars = 1 standard deviation).

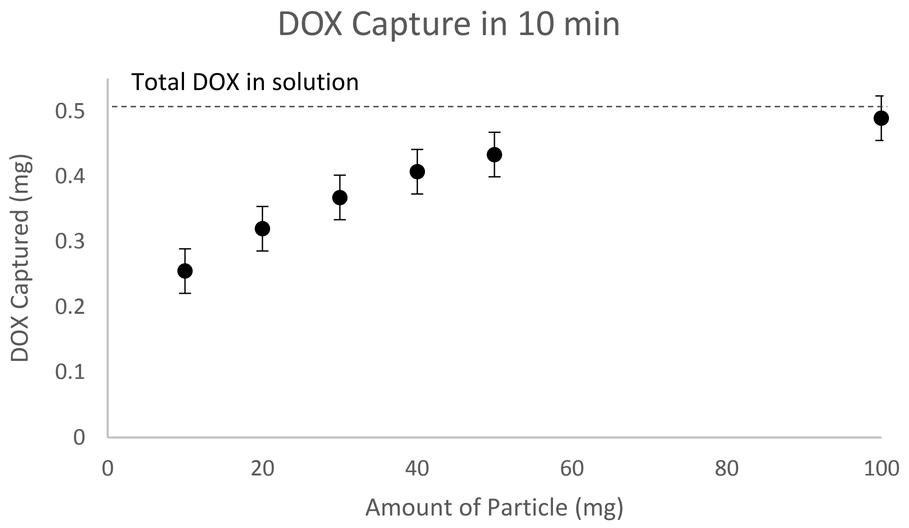

Supplementary Figure 7. DOX capture in 10 minutes from human serum (10 mL, 0.05 mg/mL, 37 °C) as a function of amount of IONP-HN3-DNA added. Average of 3 runs, error bars = 1 standard deviation.

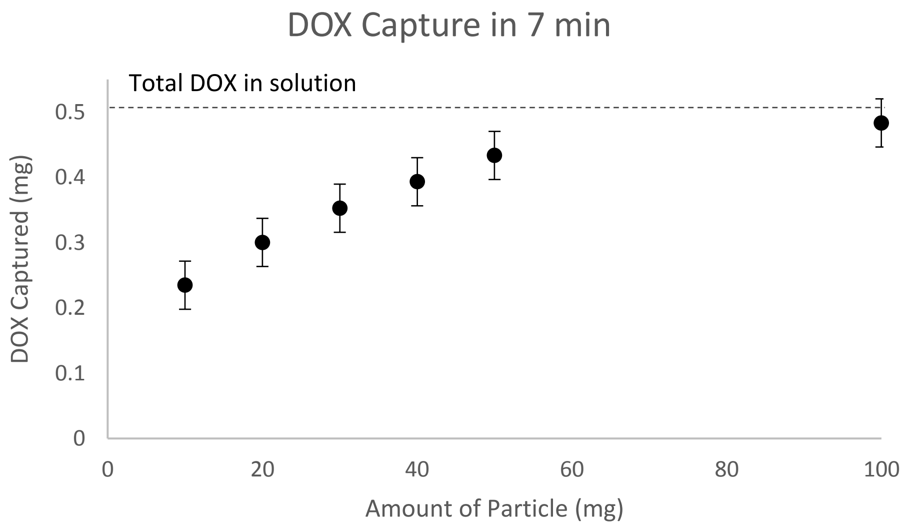

Supplementary Figure 8. DOX capture in 7 minutes from human serum (10 mL, 0.05 mg/mL, 37 °C) as a function of amount of IONP-HN3-DNA added. Average of 3 runs, error bars = 1 standard deviation.

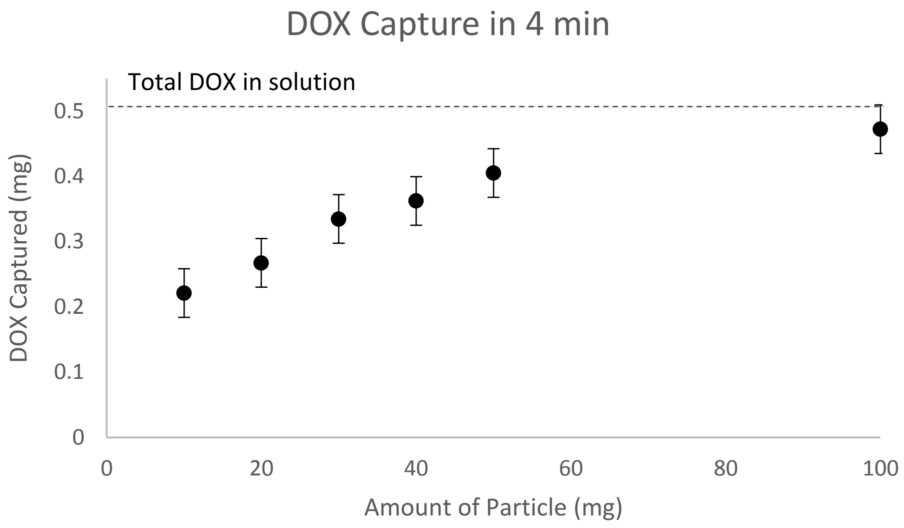

Supplementary Figure 9. DOX capture in 4 minutes from human serum (10 mL, 0.05 mg/mL, 37 °C) as a function of amount of IONP-HN3-DNA added. Average of 3 runs, error bars = 1 standard deviation.

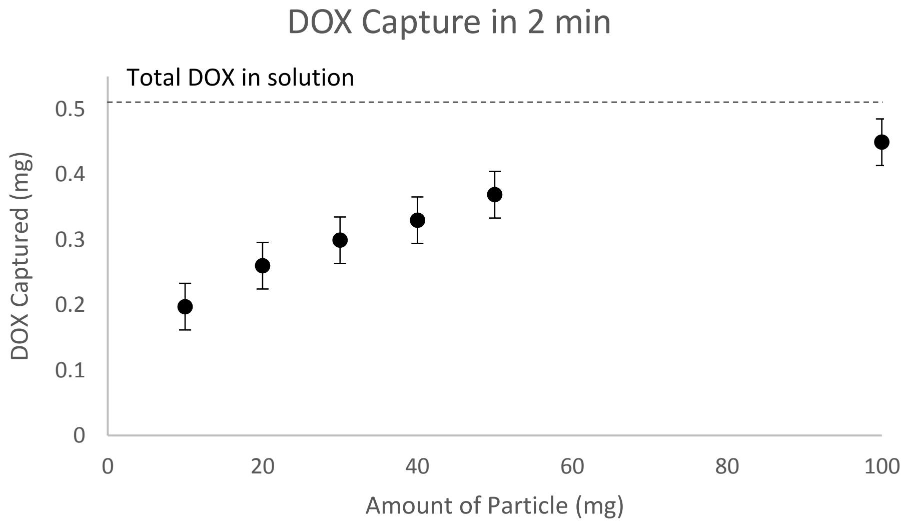

Supplementary Figure 10. DOX capture in 2 minutes from human serum (10 mL, 0.05 mg/mL, 37 °C) as a function of amount of IONP-HN3-DNA added. Average of 3 runs, error bars = 1 standard deviation.

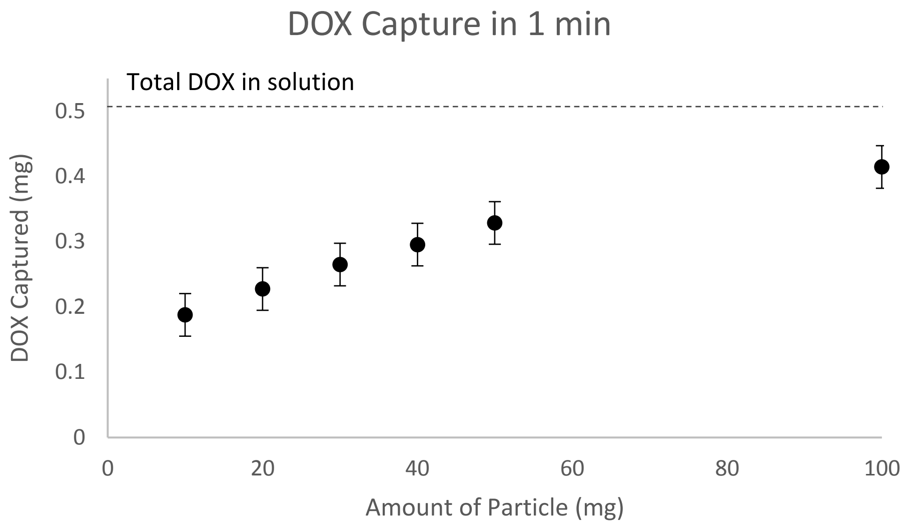

Supplementary Figure 11. DOX capture in 1 minute from human serum (10 mL, 0.05 mg/mL, 37 °C) as a function of amount of IONP-HN3-DNA added. Average of 3 runs, error bars = 1 standard deviation.

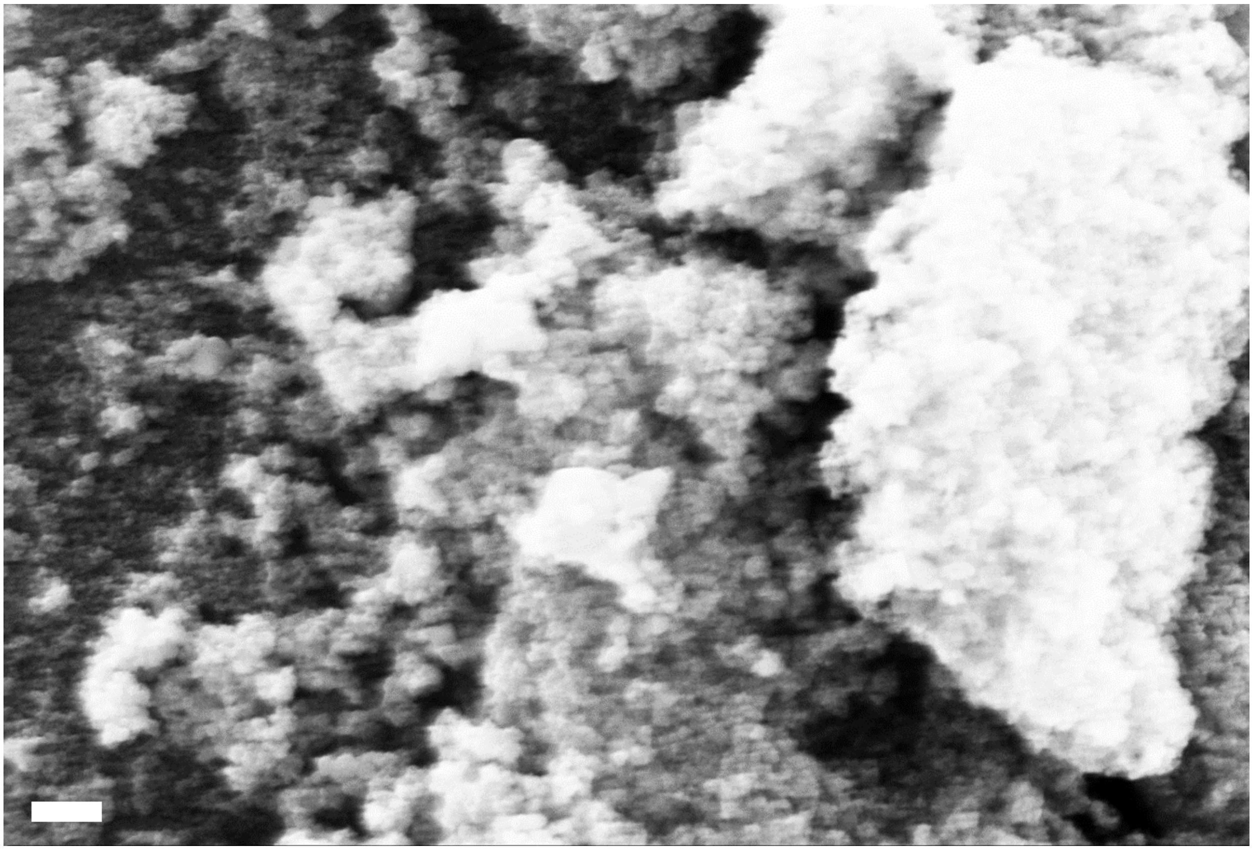

Supplementary Figure 12. SEM image of Fe_3O_4 particles. Scale bar = 200 nm.

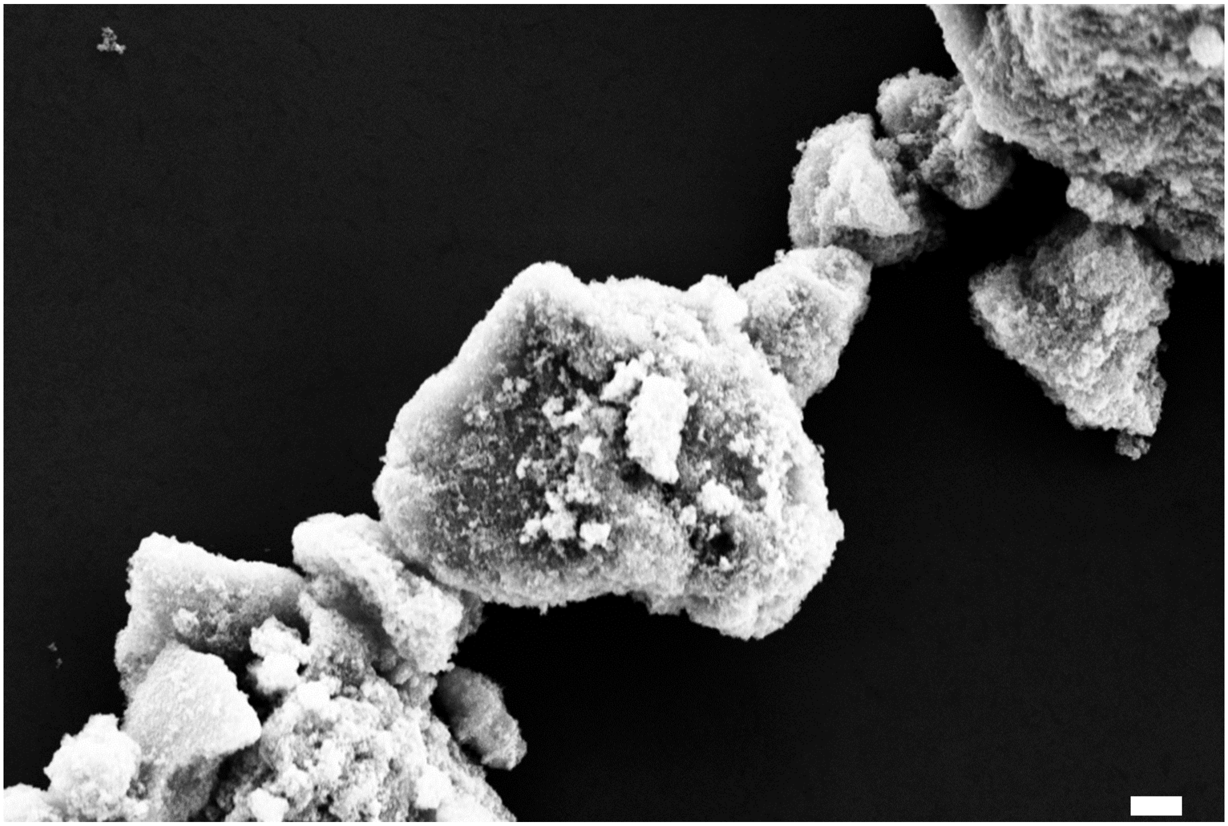
Supplementary Figure 13. SEM image of Fe_3O_4 particle aggregates. Scale bar = 1 µm.

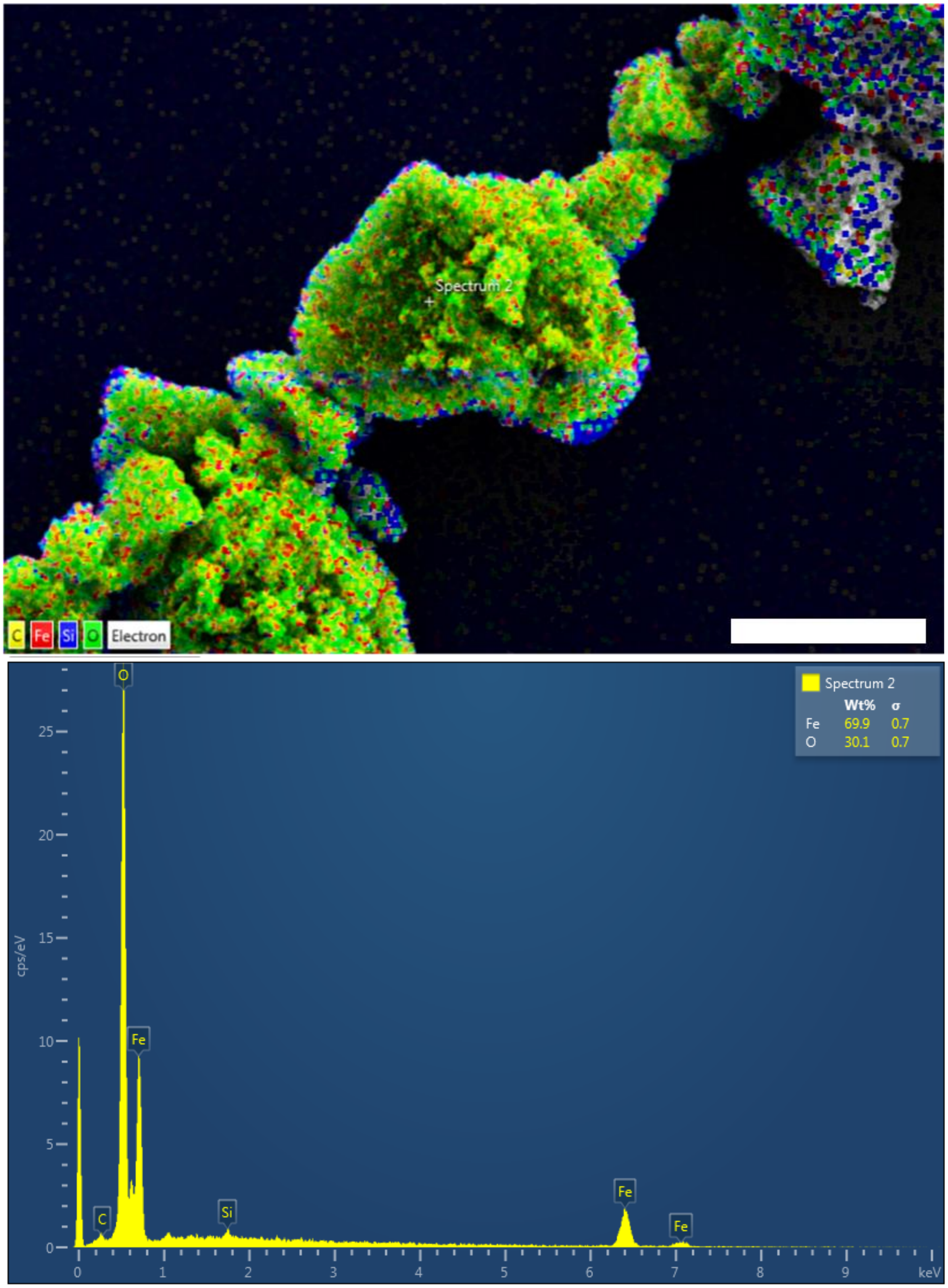

Supplementary Figure 14. EDS map and spectrum of Fe_3O_4 particle aggregates. Scale bar = 5 µm.

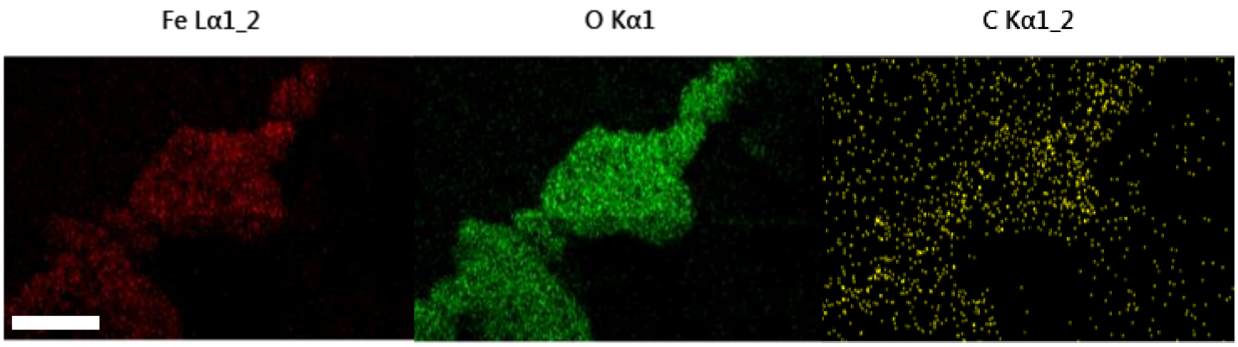

Supplementary Figure 15. EDS element maps of Fe_3O_4 particle aggregates. Scale bar = 5 µm.

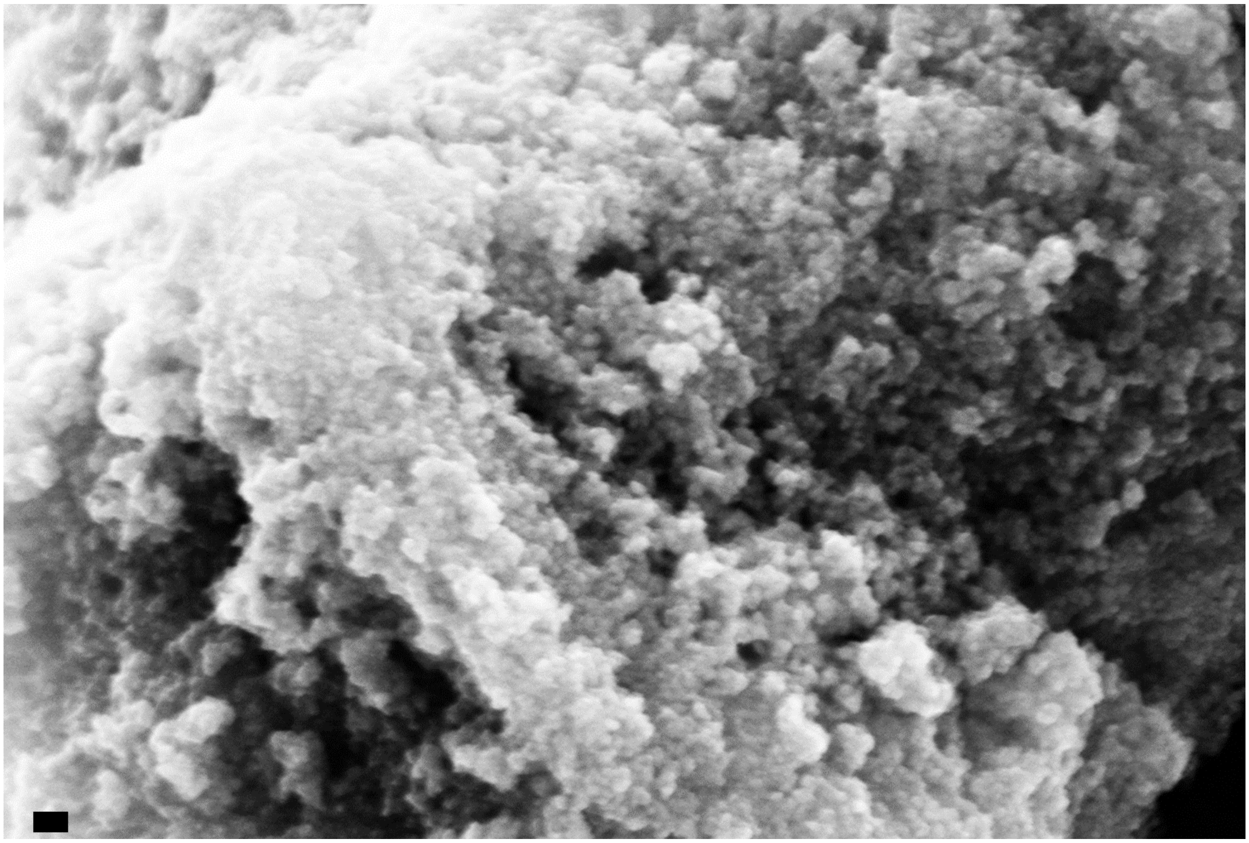
Supplementary Figure 16. SEM image of IONP-HN3-DNA. Scale bar = 100 nm.

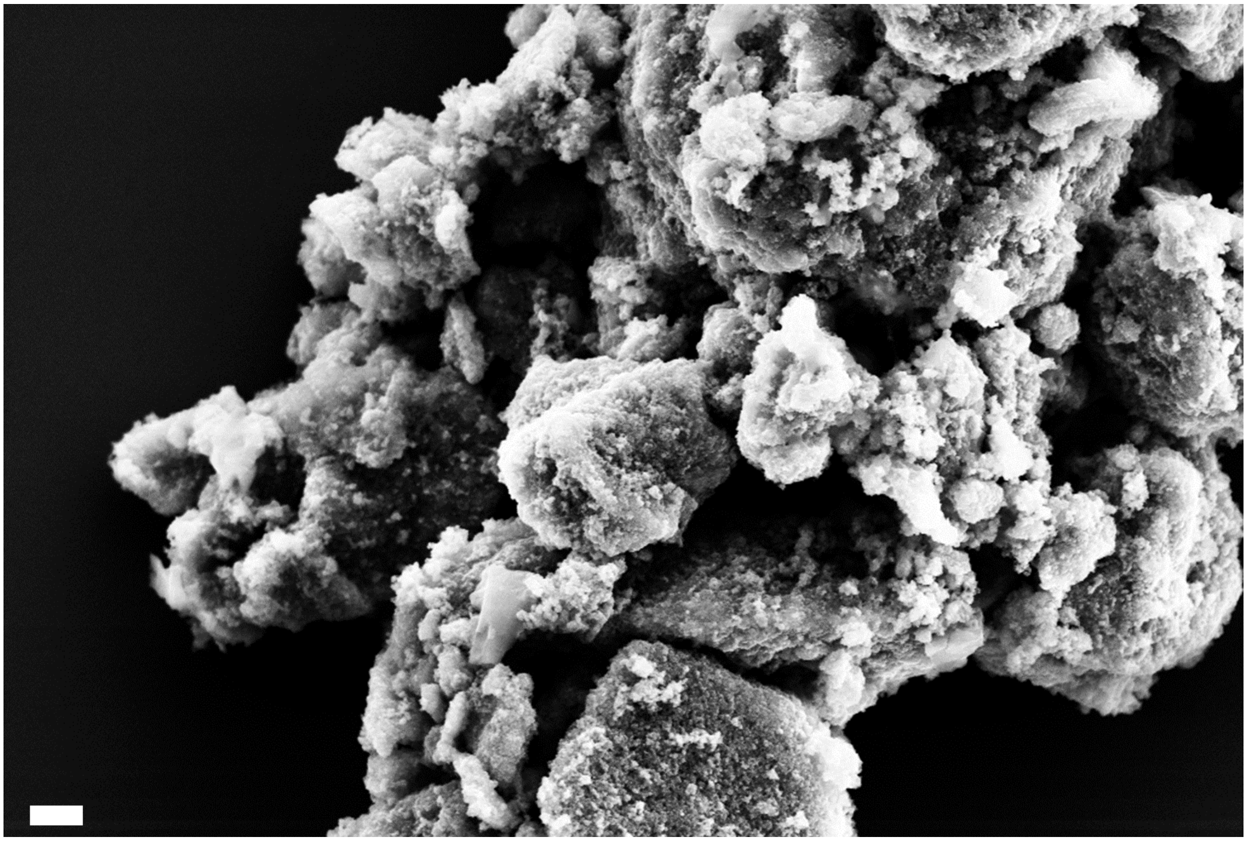

Supplementary Figure 17. SEM image of IONP-HN3-DNA aggregates. Scale bar = 1 µm.

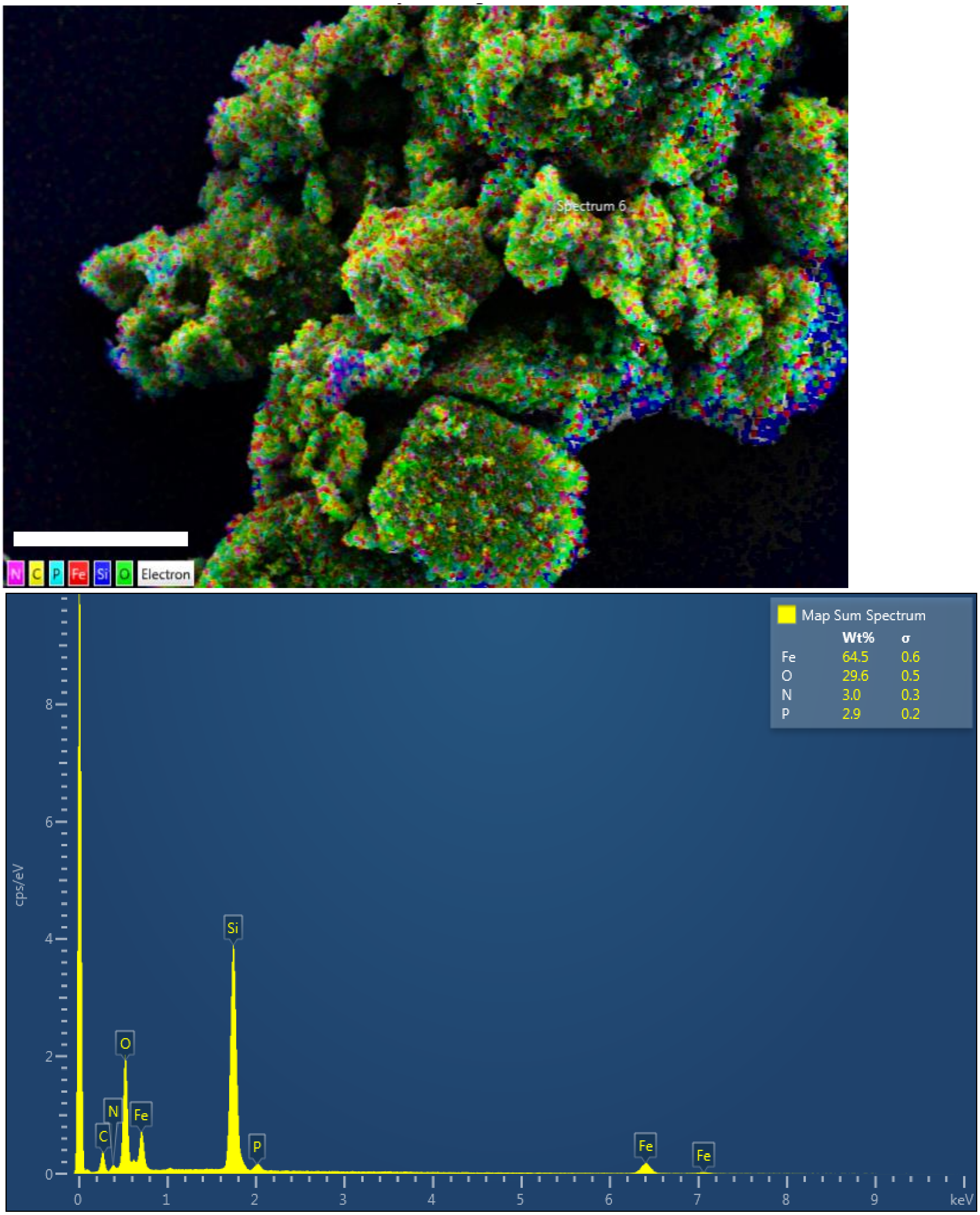

Supplementary Figure 18. EDS spectrum and map of IONP-HN3-DNA aggregates. Scale bar = 5 µm.

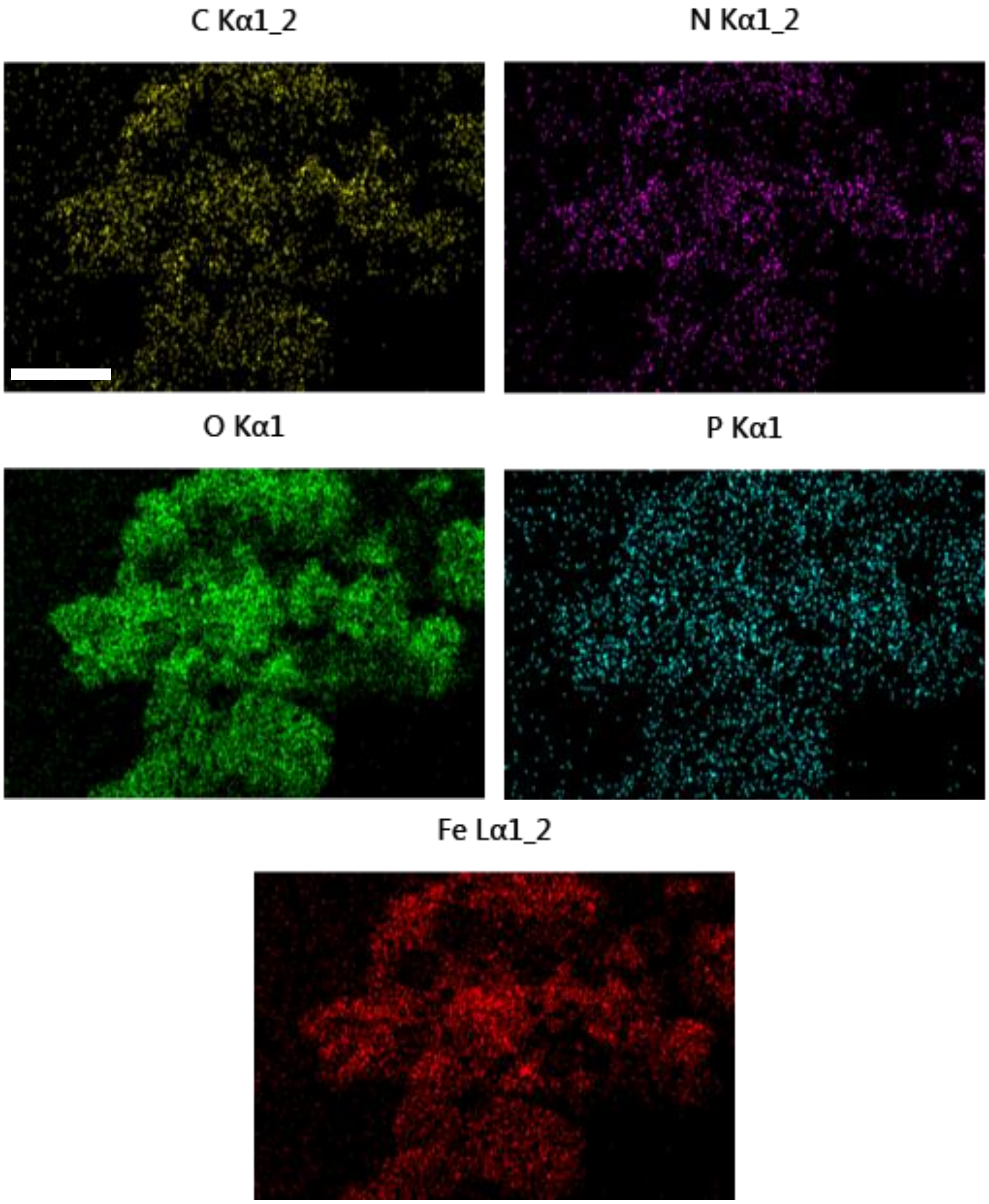

Supplementary Figure 19. EDS element maps of IONP-HN3-DNA aggregates. Scale bar = 5 µm.

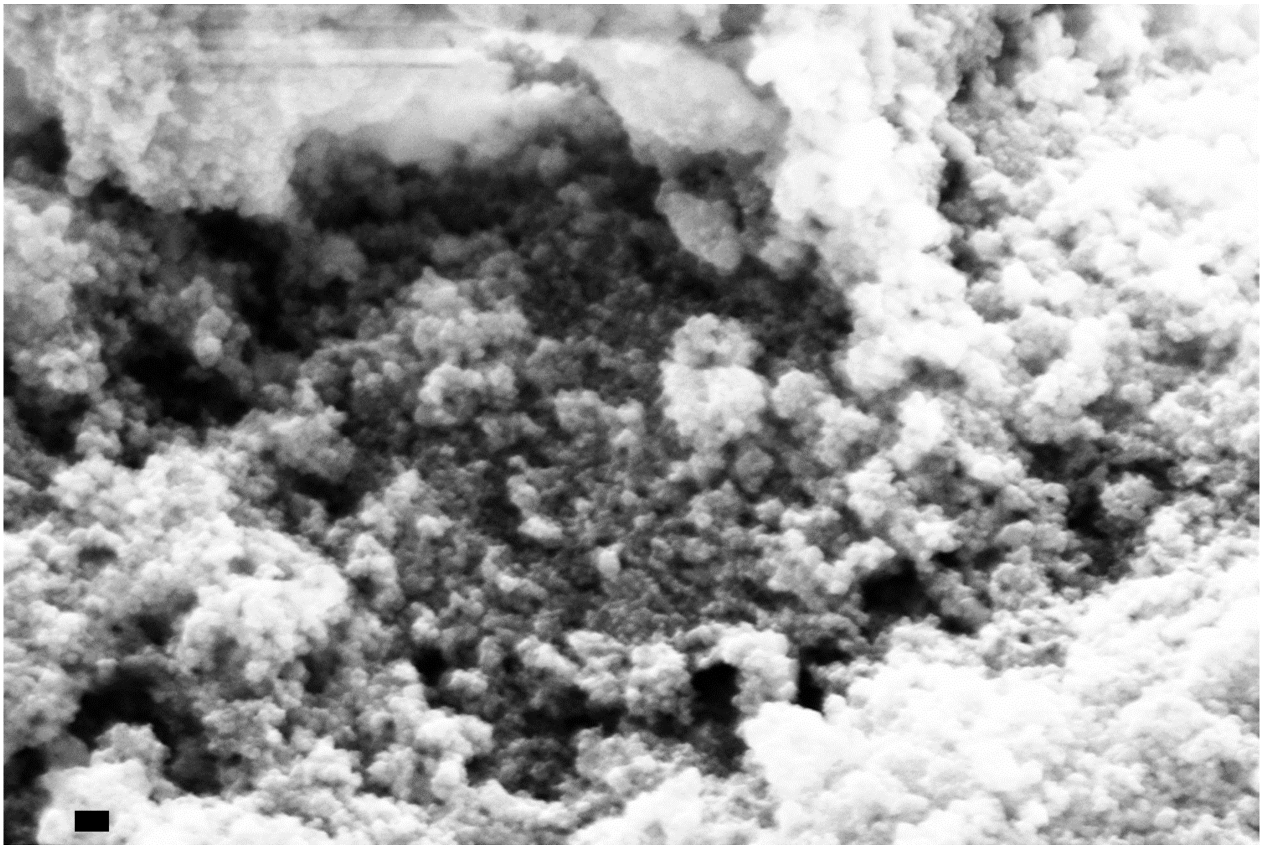

Supplementary Figure 20. SEM image of IONP-Pt-DNA. Scale bar = 100 nm.

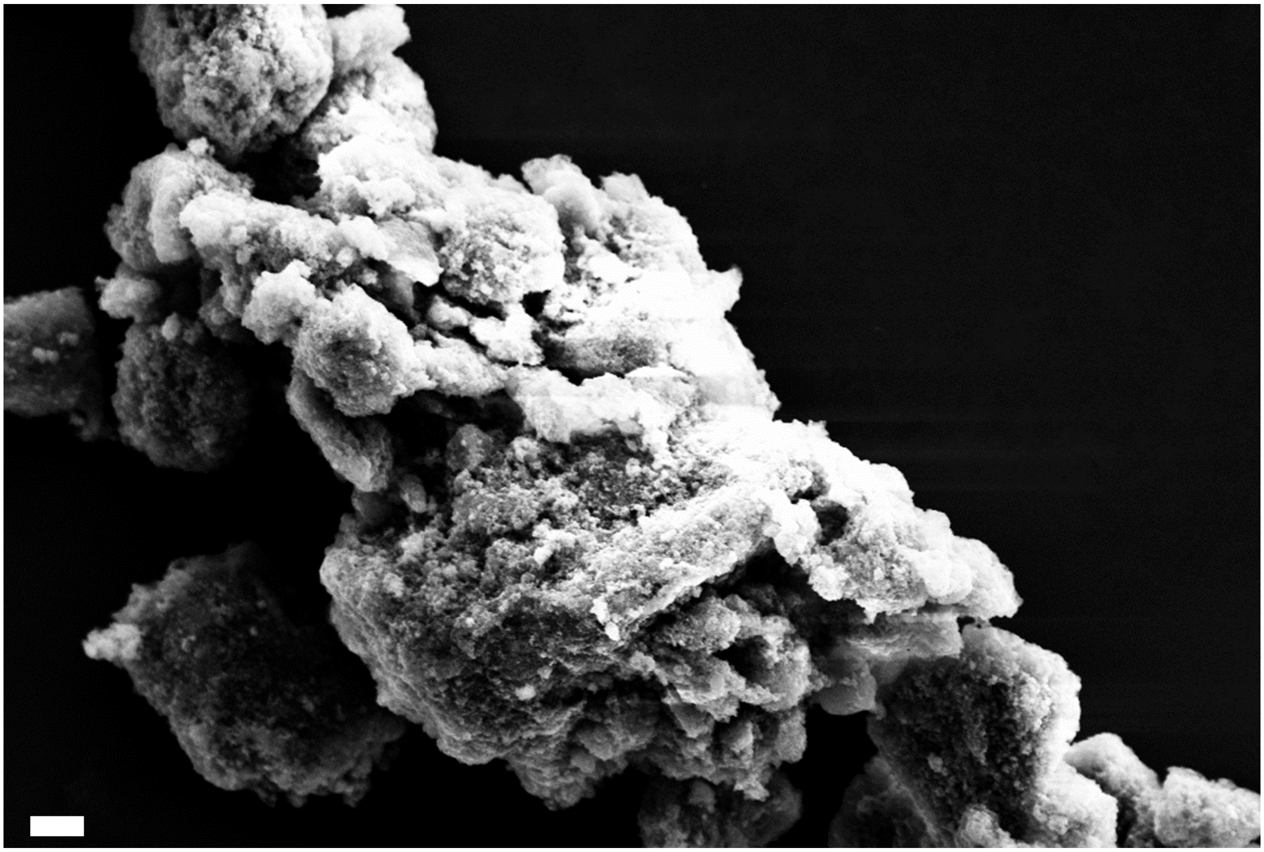

Supplementary Figure 21. SEM image of IONP-Pt-DNA aggregates. Scale bar = 1 µm.

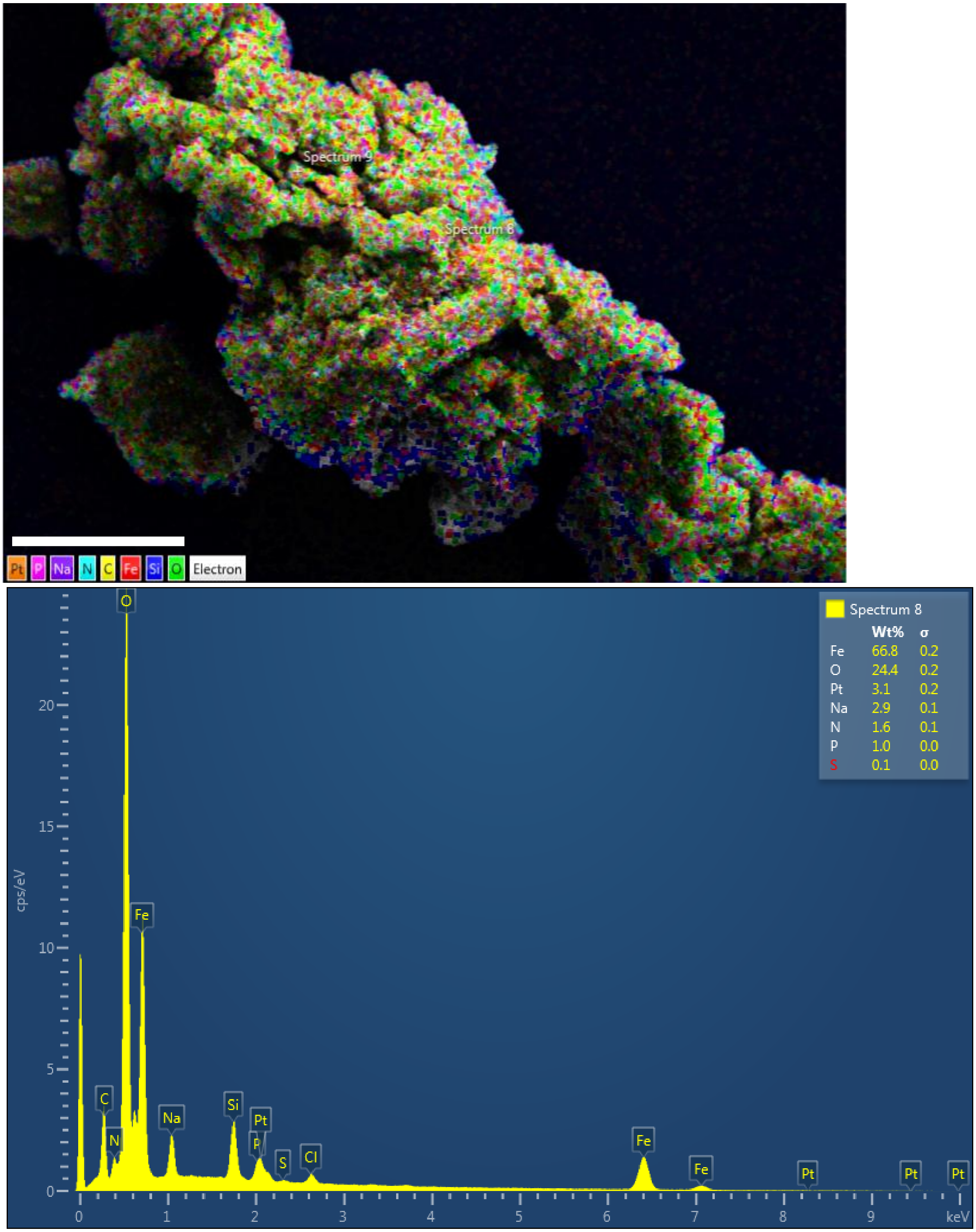

Supplementary Figure 22. EDS map and spectrum of IONP-Pt-DNA aggregates. Scale bar = 5 µm.

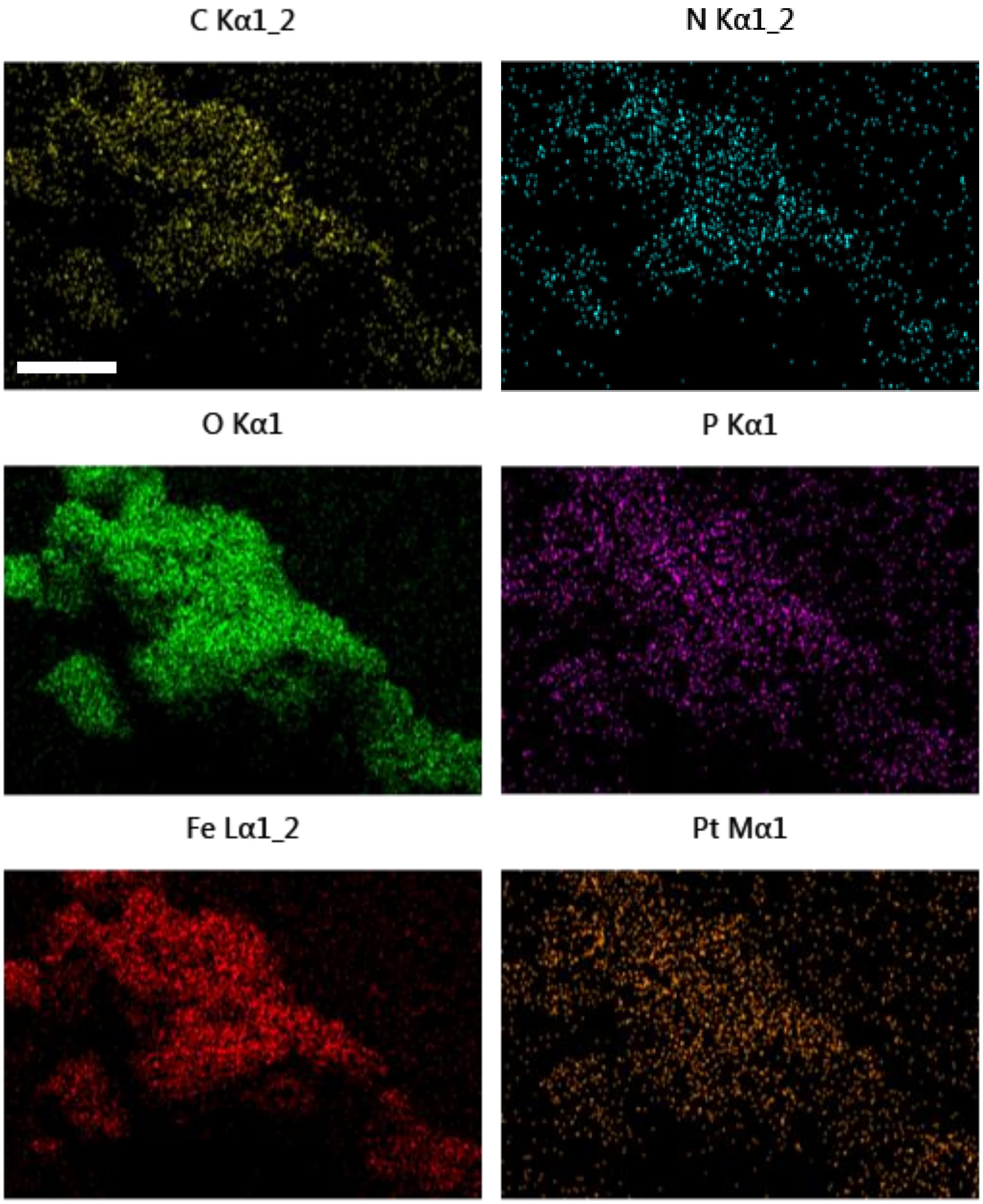

Supplementary Figure 23. EDS element maps of IONP-Pt-DNA aggregates. Scale bar = 5 µm.

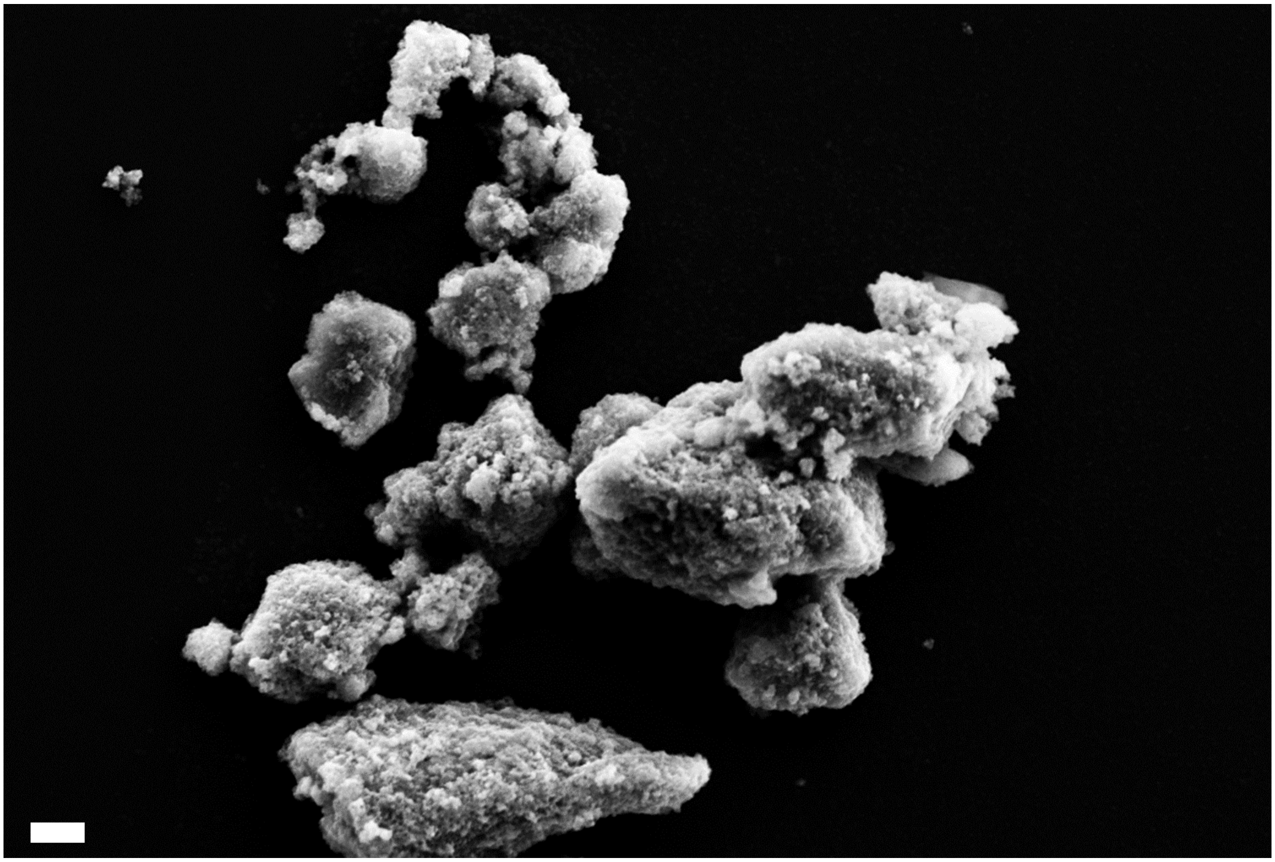

Supplementary Figure 24. SEM image of IONP-Pt aggregates. Scale bar = 1 µm.

A.

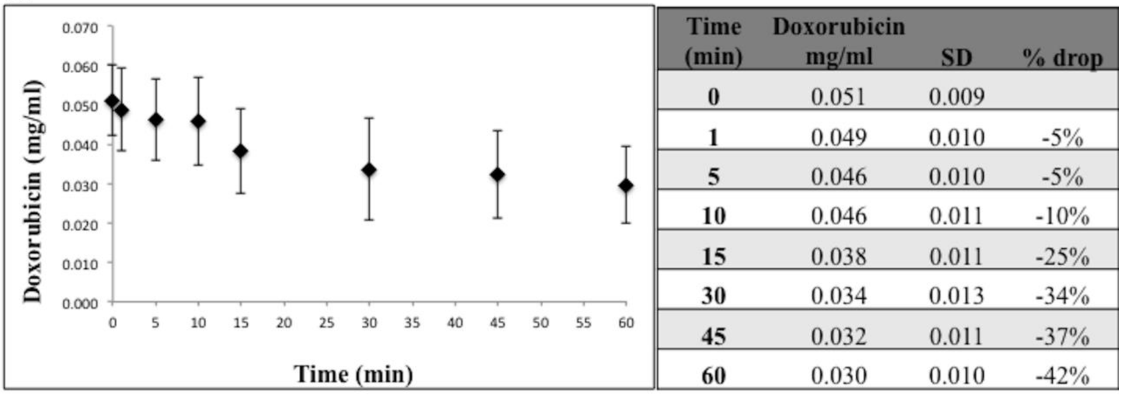

Time (min)	Doxorubicin mg/ml	SD	% drop
**0**	0.051	0.009	
**1**	0.049	0.010	-5%
**5**	0.046	0.010	-5%
**10**	0.046	0.011	-10%
**15**	0.038	0.011	-25%
**30**	0.034	0.013	-34%
**45**	0.032	0.011	-37%
**60**	0.030	0.010	-42%

B.

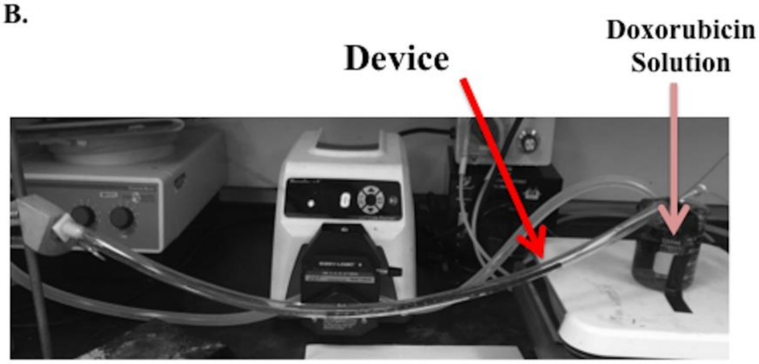

Supplementary Figure 25. A. Doxorubicin clearance from porcine blood in closed loop flow model. B. Closed loop flow model design with 25 magnet device within the lumen of the flow model. In this model, fluid flow performed the function of mechanical stirring in the earlier vial experiments. Both approaches proved sufficient to enable drug capture.

Sample ID	%C	%H	%N	Sample mass (mg)
Fe3O4	0.71	0.44	0.02	19.78
	0.44	0.37	0.02	9.267
**Average**	0.575	0.405	0.02	14.5235
**Standard Deviation**	0.19	0.05	0	-
**IONP-Pt-DNA**	5.27	1	2.15	16.98
	5.28	1.01	2.14	14.91
**Average**	5.275	1.005	2.145	15.945
**Standard Deviation**	0.007	0.007	0.007	-
**IONP-HN3-DNA**	6.6	0.99	2.64	9.933
	6.51	1.04	2.6	14.67
**Average**	6.555	1.015	2.62	12.3015
**Standard Deviation**	0.06	0.04	0.03	-
**Herring DNA**	33.81	4.61	14.5	3.666
	33.92	4.66	14.68	7.386
**Average**	33.865	4.635	14.59	5.526
**Standard Deviation**	0.08	0.04	0.13	-

Supplementary Table 1. Elemental analysis data for all materials.

Supplementary References

1. Gnapareddy, B. *et al.* Chemical and Physical Characteristics of Doxorubicin Hydrochloride Drug-Doped Salmon DNA Thin Films. *Sci. Rep.* 5, 12722 (2015).
